# Comparison of the Efficacy and Safety of General Anesthesia and Sedation Management for Esophageal Endoscopic Submucosal Dissection: A Retrospective Study

**DOI:** 10.31662/jmaj.2025-0471

**Published:** 2026-02-20

**Authors:** Susumu Yoshida, Kiyoyuki Miyasaka, Yuki Yonekura, Katsuyuki Fukuda, Seiki Abe

**Affiliations:** 1 Graduate School of Nursing Science, International University of Health and Welfare, Tokyo, Japan; 2 Department of Anesthesiology, St Luke’s International Hospital, Tokyo, Japan; 3 Graduate School of Nursing Science, St Luke’s International University, Tokyo, Japan; 4 Department of Gastroenterology, St Luke’s International Hospital, Tokyo, Japan

**Keywords:** esophagus, endoscopic submucosal dissection, general anesthesia, sedation management

## Abstract

**Introduction:**

General anesthesia (GA) for esophageal endoscopic submucosal dissection (ESD) is expected to reduce surgical complications and shorten surgical time, but definitive conclusions have not yet been reached. We retrospectively compared the efficacy and safety of GA with conventional sedation management.

**Methods:**

The study analyzed 224 consecutive cases of esophageal ESD performed at a single institution. The primary outcome was procedure time, and secondary outcomes were duration of endoscopy room stay, incidence of complications (aspiration, perforation, bleeding), and incidence of intraoperative vital sign deterioration (hypotension, bradycardia, hypoxia).

**Results:**

The study included 76 patients who underwent GA and 148 patients who underwent sedation (Sed). There was no difference in the median procedure time between the GA group, 67.5 (interquartile range [IQR], 48.8-97.8), and the Sed group, 70 (IQR, 46.0-95.25) minutes (p = 0.993). The median duration of endoscopy room stay was longer in the GA group, 121.5 (102.3-153), than in the Sed group, 90 (69-124) minutes (p = 0.000). There were no significant differences in complications, including aspiration (0/76 vs. 2/148, p = 0.550), perforation (1/76 vs. 3/148, p = 1.000), and bleeding (0/76 vs. 1/148, p = 1.000). Intraoperative vital signs showed a higher incidence of hypotension (70/76 vs. 15/148, p = 0.000) and bradycardia (34/76 vs. 10/148, p = 0.000) in the GA group.

**Conclusion:**

There was no difference in procedure time between GA and sedation management for esophageal ESD. Although complications tended to be fewer, hypotension and bradycardia were frequent with GA management. Further investigation is needed to evaluate efficacy and safety.

## Introduction

Esophageal cancer is the seventh leading cause of cancer-related mortality worldwide. It has a poor prognosis, with a five-year survival rate of only 10%-30% ^(1)^. Due to global demographic changes, the number of new cases and deaths is expected to increase significantly by 2050 ^(2)^. Asia accounts for 75% of all cases, and Japan has the fourth-highest incidence rate worldwide ^(3)^. Given this situation, the early detection and treatment of esophageal cancer in Japan are important issues. In the field of early-stage esophageal cancer surgery, endoscopic submucosal dissection (ESD), developed in Japan in the late 1990s, has brought about a revolutionary advancement. Compared with conventional endoscopic mucosal resection, ESD enables the removal of larger lesions. Evidence shows improved complete resection rates and reduced local recurrence rates. ESD is now an established minimally invasive treatment option for early-stage esophageal cancer and an alternative to surgical esophagectomy ^(4), (5)^.

Sedatives are used to mitigate patient discomfort during upper gastrointestinal endoscopic procedures. ESD, in particular, requires precise and complex maneuvers and necessitates deep sedation to ease the procedure and safely manipulate the endoscope ^(6), (7), (8)^. Due to its narrow lumen and susceptibility to the effects of heartbeat and respiration, the esophagus presents a higher procedural difficulty than the stomach, making appropriate sedation crucial. Deep sedation may inadvertently transition to general anesthesia (GA), necessitating expertise in anesthesia management and preparation ^(9)^. GA is also a treatment option, recommended for patients with complex lesions, prolonged procedures, or severe comorbidities, such as respiratory or cardiac diseases ^(10), (11)^.

GA improves procedural maneuverability by immobilizing the patient and reducing respiratory variability, and it offers high patient satisfaction ^(12)^. Although reports on GA suggest potential benefits, such as shorter procedure times, improved en bloc resection rates, and reduced complications, such as bleeding or gastrointestinal perforation, definitive conclusions have not yet been reached ^(13)^. This study aims to determine whether differences in sedation and GA management in the endoscopy room impact procedure time or complications in esophageal ESD.

## Materials and Methods

### Study design

This was a single-center retrospective observational study conducted with the approval of the Ethics Committee (St. Luke’s International University: 24-R151). Since this study had a retrospective design, written consent was waived. Consent was obtained through an opt-out method, and the opportunity to refuse participation was provided through the institutional website. Prior to September 2021, all ESD cases at the study institution were conducted in the endoscopy room under sedation by gastroenterologists. Cases requiring GA (due to severe comorbidities, cervical lesions, patient preference, etc.) were performed in the operating room under GA management by anesthesiologists. As part of ongoing hospital-wide patient safety efforts, a decision was made in September 2021 that management beyond moderate sedation in the endoscopy room should be performed by anesthesiologists. The greatest demand for anesthesia services was for upper gastrointestinal ESD procedures, all of which were scheduled under GA beyond this date.

#### Subjects and selection criteria

From consecutive cases of esophageal ESD performed at our hospital between April 2016 and December 2024, cases that met the following criteria were considered. The inclusion criteria were as follows: a single lesion, age 18 years or older, cases performed in the endoscopy room, and cases performed under sedation or GA. The exclusion criteria were as follows: cases performed in the operating room, cases performed concurrently with other surgical procedures, and cases with incomplete data or insufficient records.

#### GA and sedation management

In the GA group, anesthesiologists were in charge, and drug selection and dosage were determined at their discretion. After induction of anesthesia in the supine position, the patient was repositioned to the left lateral position, and the procedure was started. Induction was performed using intravenous anesthetics (propofol or remimazolam), remifentanil, and rocuronium, and after tracheal intubation, mechanical ventilation was performed using an anesthesia machine. Anesthesia maintenance was performed using remifentanil and intravenous anesthetics (propofol or remimazolam) or volatile inhalation anesthetics (sevoflurane or desflurane). An appropriate depth of anesthesia was maintained during the operation using electroencephalogram monitoring or exhaled anesthetic gas concentration. At the time of anesthesia emergence, adequate recovery from muscle relaxation was confirmed using a neuromuscular monitor, and the endotracheal tube was removed. Postoperative analgesia was administered with fentanyl or acetaminophen at the discretion of the anesthesiologist. After discharge from the endoscopy room, oxygen at 6 L/min was administered via a face mask for two hours. Vital signs in the endoscopy room were recorded as continuous data in the anesthesia record.

In the sedation group, gastroenterologists administered midazolam and analgesics (pethidine or pentazocine), targeting moderate to deep sedation. After entering the endoscopy room, the patient was placed in the left lateral position, and the medication was administered before the procedure began. Drug selection, dosage, and timing of additional administration were determined by the gastroenterologists. If sedation was insufficient despite multiple administrations of midazolam and analgesics, haloperidol was added. Oxygen was administered at a rate of 2 L/min via a nasal cannula during the procedure and discontinued at the end. Patients who experienced a decrease in oxygen saturation after discontinuation of oxygen continued to receive oxygen in the ward. During sedation, a separate qualified individual monitored the patient, and vital signs were recorded every five minutes.

Following GA or sedation, patients were discharged directly from the endoscopy room to the inpatient ward once they met institutional recovery criteria. If patients did not meet recovery criteria, they were transferred to the intensive care unit for further observation and evaluation.

### Endpoints

#### Primary endpoint

Procedure time (time from endoscope insertion to final removal).

#### Secondary endpoints

Duration of endoscopy room stay (time from entering to leaving the room), complications (aspiration pneumonia, bleeding, gastrointestinal perforation), and intraoperative vital sign deterioration events (hypotension: systolic blood pressure below 90 mmHg; bradycardia: heart rate <50 bpm; desaturation: oxygen saturation below 90%).

### Definition of complications

Aspiration pneumonia: clinical diagnosis or imaging diagnosis, including intraoperative findings or suspected pneumonia due to postoperative fever; Bleeding: blood transfusion or postoperative hemostasis measures; Gastrointestinal perforation: intraoperative findings or postoperative imaging diagnosis.

### Statistical analysis

Descriptive statistics were presented as the mean (± standard deviation) for continuous variables that followed a normal distribution and as the median (interquartile range [IQR]) for those that followed a non-normal distribution. Patient numbers were presented as counts and percentages (%). For comparisons between two groups, *t*-tests were used for continuous variables that followed a normal distribution, and Mann-Whitney U tests were used for non-normal distributions. Fisher’s exact test or chi-square tests were used for categorical data. To compare the procedure time between GA and sedation after adjusting for confounding factors, multiple regression analysis was performed using body mass index, lower esophageal lesions ^(14)^, lesions with a length of 30 mm or more ^(14)^, and alcohol consumption as covariates. The threshold for statistical significance was set at p < 0.05. Statistical analysis was performed using IBM^Ⓡ^ SPSS^Ⓡ^ Statistics Ver. 29.

## Results

**Figure 1.**
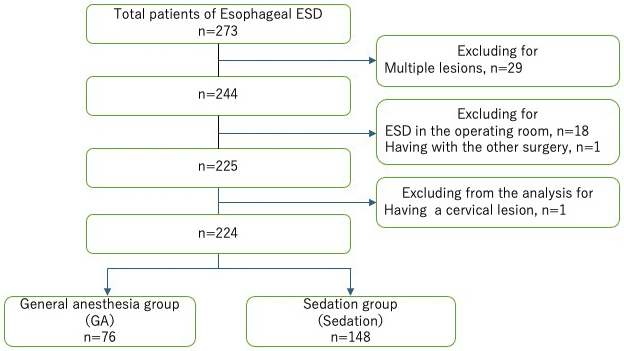
Flow diagram of participants through the study.

During the study period, a total of 273 cases of esophageal ESD were performed. Of this total, 225 cases were included, and one patient with a cervical lesion was excluded from the final analysis. They divided into a GA group of 76 patients and a sedation group of 148 patients (Figure 1). In terms of patient characteristics between the two groups (Table 1), the median lesion diameter (length) was 26 mm (IQR: 20-32.8) vs. 30 mm (IQR: 23.8-40), p = 0.008), and the median lesion area was 528 mm^2^ (IQR: 303.8.-734) vs. 710 mm^2^ (IQR: 419-1,255.5), p = 0.003. Anesthetics and sedatives used in the GA group were primarily propofol (41 patients, 53.9%) and desflurane (31 patients), whereas in the sedation group, midazolam (148 patients, 100%) and meperidine (143 patients, 96.6%) were used (Table 2). The primary endpoint, median procedure time, was 67.5 minutes (IQR: 48.8-97.8) in the GA group and 70 minutes (IQR: 46.0-97.3) in the sedation group, with no significant difference between the two groups (p = 0.993). The secondary endpoint, median endoscopy room stay time, was 121.5 minutes (IQR: 102.3-153) in the GA group and 90 minutes (IQR: 69-124) in the sedation group, with the GA group significantly longer (p < 0.001). There were no significant differences between the two groups in terms of complications such as aspiration pneumonia (0 patients [0%] vs. 2 patients [1.3%], p = 0.550), bleeding (0 patients [0%] vs. 1 patient [1%], p = 1.000), and gastrointestinal perforation (1 patient [1.3%] vs. 3 patients [2%], p = 1.000). Intraoperative vital sign deterioration events were significantly more common in the GA group for hypotension (70 patients [92.1%] vs. 15 patients [10.1%], p < 0.001) and bradycardia (34 patients [44.7%] vs. 10 patients [6.8%], p < 0.001), whereas there was no significant difference between the two groups in terms of desaturation (1 patient [1%] vs. 1 patient [1%], p = 1.000) (Table 3). The results of multivariate regression analysis showed a significant difference in lesion size (30 mm or larger) (p < 0.001, Table 4).

**Table 1.** Patient Characteristics.

Variables	GA group (n = 76)	Sedation group (n = 148)	p Value	
Age (years), mean ± SD	66.7 ± 10.0	69.1 ± 10.4	0.090	a
Sex (male/female), n (%)	58 (76.3) /18 (23.4)	121 (81.8) / 27 (18.2)	0.336	b
Height (cm), mean ± SD	167.9 ± 7.8	166.7 ± 7.5	0.257	a
Weight (kg), mean ± SD	64.6 ± 12.4	64.8 ± 11.6	0.936	a
BMI (kg/m^2^), mean ± SD	19.2 ± 3.1	19.4 ± 3.0	0.631	a
Location of lesion (upper/middle/lower), n (%)	11 (14.5)/46 (60.5)/19 (25)	21 (14.2)/82 (55.4)/45 (30.4)	0.688	b
Lesion area (length × width) in mm^2^, median (IQR)	528 (303.8, 734)	710 (419, 1255.5)	0.003	c
Lesion diameter (length) in mm, median (IQR)	26 (20, 32.8)	30.0 (23.8, 40)	0.008	c
Hypertension, n (%)	41 (53.9)	77 (52.0)	0.785	b
Diabetes mellitus, n (%)	10 (13.2)	27 (18.2)	0.332	b
Dyslipidemia, n (%)	15 (19.7)	32 (21.6)	0.743	b
Liver dysfunction, n (%)	9 (11.8)	23 (15.5)	0.547	b
Heart disease, n (%)	14 (18.4)	26 (17.6)	0.856	b
Chronic kidney disease, n (%)	22 (28.9)	29 (19.6)	0.131	b
Daily alcohol intake, n (%)	62 (82.6)	115 (77.7)	0.500	b
Smoking, n (%)	37 (42.1)	85 (57.4)	0.213	b
Preoperative systolic blood pressure (mmHg), mean ± SD	124.7 ± 13.4	124.7 ± 15.0	0.986	a
Preoperative heart rate (bpm), mean ± SD	70.6 ± 10.3	68.9 ± 10.5	0.257	a
ASA-PS (1/2/3), n (％)	3 (4)/68 (89.5)/5 (6.6)	18 (12.2)/120 (81.1)/10 (6.8)	0.133	b

ASA-PS: American Society of Anesthesiologists performance status; bpm: beat per minute; BMI: body mass index; IQR: interquartile range; SD: standard deviation. a; t-test, b; chi-square, c; Mann-Whitney U test.

**Table 2.** Anesthetics and Sedatives.

Variables	GA group（n = 76）	Sedation group（n = 148）	p Value	
General anesthesia				
Propofol (maintenance), n (%)	41 (53.9)	NA	NA	
Remimazolam, n (%)	2 (2.6)	NA	NA	
Sevoflurane, n (%)	2 (2.6)	NA	NA	
Desflurane, n (%)	31 (40.8)	NA	NA	
Remifentanil, n (%)	76 (100)	NA		
Sedation				
Midazolam, n (%)	NA	148 (100)	NA	
Meperidine (pethidine), n (%)	NA	143 (96.6)	NA	
Pentazocine, n (%)	NA	5 (3.5)	NA	
Haloperidol, n (%)	NA	3 (2)	NA	
Others				
Vasodilator, n (%)	0 (0)	6 (4)	0.098	a
Vasopressor, n (%)	69 (90.8)	0 (0)	<0.001	a
Scopolamine, n (%)	11 (14.8)	107 (72.5)	<0.001	b
Glucagon, n (%)	4 (5.3)	18 (12.1)	0.153	a
Flumazenil, n (%)	2 (2.6)	2 (1.3)	0.606	a

NA: not available. a; Fisher’s exact test, b; chi-square test.

**Table 3.** Surgery Outcomes.

Variables	GA group (n = 76)	Sedation group（n = 148)	p Value	
**Primary outcome**				
Operation time (min), median (IQR)	67.5 (48.8, 97.8)	70.0 (46.0, 97.3)	0.993	a
**Secondary outcomes**				
Endoscopy room stay time (min), median (IQR)	121.5 (102.3, 153)	90 (69, 124)	<0.001	a
Time from entering the room to the start of operation (min), median (IQR)	24 (22, 27.3)	12 (9, 16)	<0.001	a
Time from operation completion to discharge (min), median (IQR)	28 (25, 32.3)	6 (5, 10)	<0.001	a
Aspiration pneumonia, n (%)	0 (0)	2 (1.3)	0.550	b
Bleeding, n (%)	0 (0)	1 (1)	1.000	b
Perforation, n (%)	1 (1.3)	3 (2)	1.000	b
Hypotension, n (%)	70 (92.1)	15 (10.1)	<0.001	c
Bradycardia, n (%)	34 (44.7)	10 (6.8)	<0.001	c
Desaturation, n (%)	1 (1.3)	1 (1)	1.000	b
Oxygen administration after ESD, n (%)	76 (100)	6 (4)	<0.001	c
Resection speed (Lesion size/Operation time, mm^2^/min), mean ± SD	8.5 ± 4.1	11.1 ± 5.2	<0.001	d
Postoperative pain (NRS), median (IQR)	0 (0, 4)	0 (0, 3)	0.192	a
Postoperative nausea or vomiting, n (%)	2 (2.6)	9 (6)	0.341	b
Unexpected ICU admission, n (%)	0 (0)	1 (1)	1.000	b

ICU: intensive care unit; IQR: interquartile range; NRS: numerical rating scale. a; Mann-Whitney U test, b; Fisher’s exact test, c; chi-square test d; t-test.

**Table 4.** Multivariable Regression Analysis for Operation Time in Esophageal ESD.

Variables	B	Standard error	95% confidential interval	p Value
GA or Sedation, GA = 1, Sedation = 0	1.513	4.929	−8.20 to 11.23	0.759
BMI	0.809	0.775	−0.72 to 2.34	0.298
Lesion of location (lower), Yes = 1, No = 0	1.061	5.300	−9.39 to 11.51	0.841
Lesion size (≧30mm), Yes = 1, No = 0	37.319	4.753	27.91-46.73	<0.001
Daily alcohol intake, Yes = 1, No = 0	−8.205	5.746	−19.53 to 3.12	0.155

BMI: body mass index; ESD: endoscopic submucosal dissection; GA: general anesthesia.

## Discussion

To the best of our knowledge, this is the largest comparative study of anesthetic methods for patients undergoing esophageal ESD. In this study, no difference in procedure time was observed between the GA group and the sedation group. Additionally, though there was a tendency toward fewer complications such as bleeding, gastrointestinal perforation, and aspiration pneumonia in the GA group, no statistically significant difference was noted. In the GA group, there were more instances of hypotension and bradycardia during surgery, and the endoscopy room stay time was significantly longer.

The primary endpoint for this study was procedural time. We posited that the overall procedure time would be shortened under GA, since the procedure does not need to be interrupted to respond to changes in vital signs, airway issues, as well as patient movement or interactions. In comparisons of procedure times between the two groups, previous studies have reported conflicting results. Rong et al. ^(12)^ reported that in a mixed cohort of gastric and esophageal ESD, procedure time was shorter in the GA group. Subsequent reports have also suggested that resection speed was faster in the GA group ^(15)^, while other reports have indicated no difference or longer procedure times ^(16), (17)^. In this study, the sedation group had larger lesion sizes and faster resection speeds.

Factors influencing procedure time include lesion location, size, and circumference ^(18)^. Lesion size is closely associated with the technical difficulty of esophageal ESD ^(19)^, and tumors exceeding 30 mm in size have been reported as an independent predictor of difficult esophageal ESD cases associated with prolonged procedure time (over 120 minutes) (odds ratio [OR]: 9.17, p < 0.001) ^(20)^. This is consistent with the results of this study. Although it has been pointed out that lesion location, particularly lower or left-wall lesions, is associated with higher resection difficulty ^(21)^, no significant effect of lesion location was observed in this study. One cervical case was excluded from the analysis, and its impact on the results may be minimal. Additionally, while it has been noted that a circumference of one-half or more may influence procedure time ^(14), (18)^, this study did not examine the impact of the lesion circumference. When considering the relationship between procedure time and resection speed, factors not measured in this study, such as surgeon skill ^(22)^ or advancements in equipment and techniques, may have influenced the results ^(23)^.

The main complications of esophageal ESD include aspiration, perforation, mediastinal emphysema, bleeding, and stricture. Compared to gastric or colorectal ESD, esophageal ESD is technically more challenging due to anatomical factors (absence of serosa, thin muscular layer, and significant influence from cardiac contractions and respiratory movements). Even slight patient movement can easily cause muscular layer damage, necessitating appropriate sedation. GA offers several benefits in reducing complications, including the ability to perform the procedure without interruption due to immobilization ^(24)^, reduced aspiration risk through airway separation via a tracheal tube ^(16)^, and allowing the surgeon to focus on the procedure due to anesthesiologist monitoring. The complication rates are as follows: perforation occurs in approximately 1.5%-5.0% of cases ^(4), (25)^, aspiration occurs in approximately 1.6%-1.7% of cases ^(23), (26)^, and bleeding occurs in approximately 0.6%-2.1% of cases ^(26), (27)^, with all rates being low. In this study, two cases of aspiration occurred due to vomiting during surgery in the sedation group, but none occurred in the GA group. Although no statistical difference was observed, the benefits of GA are considered to have been obtained. The absence of a difference in the incidence of perforation was in contrast to multiple previous studies. Kim et al. ^(16)^ reported that no acute procedure-related complications (perforation, postoperative bleeding, cardiopulmonary events, new pulmonary infiltrates, etc.) occurred in the GA group, whereas 9.6% occurred in the sedation management group, recommending GA as a safe option. Other reports also state that GA was associated with no perforations or aspiration pneumonia and fewer adverse events ^(10), (15), (17)^. On the other hand, Yagi Kuwata et al. ^(22)^ reported that the sedation group had shorter procedure times and significantly lower complication rates, but noted in their discussion that the higher number of experienced operators in the GA group may have confounded the results. The findings that showed no significant differences between the two groups may be due to the low incidence of complications in both groups, making it difficult to detect statistical significance.

A systematic review comparing outcomes of upper gastrointestinal ESD in the GA and sedation management groups in 2023 ^(13)^ concluded that the en bloc resection rate was significantly higher in esophageal ESD (five studies, 518 vs. 495 patients), with a trend toward reduced complications such as procedure time, gastrointestinal perforation, bleeding, and aspiration pneumonia. Further accumulation of research and the implementation of larger-scale, higher-quality studies are required in this area (only one prospective study has been conducted).

Intraoperative vital sign deterioration showed that the incidence of hypotension and bradycardia was significantly higher in the GA group. These findings are consistent with the pharmacological effects of general anesthetics. Recent evidence suggests that intraoperative hypotension (mean arterial pressure <60 to 70 mmHg or systolic blood pressure <90 to 100 mmHg) may increase the risk of myocardial injury, renal failure, and mortality, and avoidance of intraoperative hypotension is recommended ^(28)^. In this study, during GA, anesthesiologists managed blood pressure and heart rate according to target values set for each patient. Although the incidence of hypotension and bradycardia was high, no cerebral or cardiopulmonary complications other than aspiration pneumonia were observed during the hospitalization period. However, the impact of hypotension on long-term complications such as postoperative delirium and cognitive impairment remains unclear ^(29), (30)^. Hypotension was defined as systolic blood pressure <90 mmHg ^(31)^, but adopting mean arterial pressure or other cutoff values is unlikely to significantly affect these results. Remimazolam, a newly approved anesthetic, has been reported to have greater hemodynamic stability compared to propofol (the incidence of hypotension was 32.1% vs. 67.9%) ^(32)^. If remimazolam was frequently used in the GA group, it may have influenced the results. Abbott et al. ^(33)^ revealed that, in addition to abnormal blood pressure during surgery, tachycardia (heart rate <100 bpm) significantly increased myocardial injury (OR: 1.27, p < 0.01), while bradycardia (heart rate <55 bpm) reduced it (OR: 0.70, p < 0.01), indicating that the impact of bradycardia on adverse events was minimal. In sedation management, particular attention should be paid to respiratory-related complications such as respiratory depression or apnea ^(9), (34)^. The fact that no special intervention was required for the single case of desaturation may be attributed to the effects of oxygen administration during the operation and continuous monitoring of consciousness and respiratory status.

### Limitations of the study

Although this is a retrospective design, it is a comparison between two periods, and it is assumed that the patient backgrounds of the two groups are uniform; therefore, propensity score matching was not performed. However, the presence of confounding factors that may affect the results, such as lesion size, is a concern. Factors that could influence the difficulty of the procedure, such as lesion scars, the presence of varicose veins, the endoscopist’s skill level, and the use of antiplatelet drugs, were not evaluated in this study ^(35), (36)^. Additionally, the choice of medication and dosage was left to the physician’s discretion, and there were no records of sedation levels, blood pressure measurement sites, frequency of body movement, or procedural interruptions in the medical records, which may have influenced the results. Although statistical power may have been insufficient to detect surgical complications, there was no difference in procedure time, with an apparent decrease in procedural speed and prolonged endoscopy room stays with GA. From an economic perspective, GA may be considered for cases in which procedural difficulty is anticipated ^(36)^. However, GA offers useful benefits, including the avoidance of aspiration and higher patient and endoscopist satisfaction ^(12)^. The results of this study also indicate that the incidence of complications tends to be low under GA. It is necessary to consider sedation methods not only in terms of procedural outcomes but also in terms of qualitative effects on patients and endoscopists.

In conclusion, in a comparison of GA and sedation management in esophageal ESD, there was no significant reduction in procedure time. In the GA group, complications tended to be fewer, but intraoperative hypotension and bradycardia were frequent. Going forward, it is necessary to conduct large-scale, high-quality studies, validate qualitative effects on patients and endoscopists, and comprehensively consider anesthesia and sedation management methods.

## Article Information

### Author Contributions

Susumu Yoshida designed the study, collected data, analyzed and interpreted the data, and wrote the manuscript in a major contribution. Kiyoyuki Miyasaka, Katsuyuki Fukuda, and Yuki Yonekura aided in interpreting the results and worked on the manuscript. Seiki Abe supervised the project. Susumu Yoshida wrote the manuscript with support from Kiyoyuki Miyasaka. All the authors critically reviewed the manuscript and approved the final version.

### Conflicts of Interest

None

### IRB Approval Code and Name of Institution

St. Luke’s International Hospital (24R-151).
